# Rapid Detection of Single- and Co-Contaminant Aflatoxins and Fumonisins in Ground Maize Using Hyperspectral Imaging Techniques

**DOI:** 10.3390/toxins15070472

**Published:** 2023-07-22

**Authors:** Yong-Kyoung Kim, Insuck Baek, Kyung-Min Lee, Geonwoo Kim, Seyeon Kim, Sung-Youn Kim, Diane Chan, Timothy J. Herrman, Namkuk Kim, Moon S. Kim

**Affiliations:** 1 Division of Safety Analysis, Experiment & Research Institute, National Agricultural Products Quality Management Service, Gimcheon 39660, Republic of Korea; ykkim79@korea.kr (Y.-K.K.); syesther1114@korea.kr (S.K.); youn5326@korea.kr (S.-Y.K.); 2 Environmental Microbial and Food Safety Laboratory, Agricultural Research Service, U.S. Department of Agriculture, Powder Mill Rd., Building 303 BARC-East, Beltsville, MD 20705, USA; insuck.baek@usda.gov (I.B.); geonwookim@gnu.ac.kr (G.K.); diane.chan@usda.gov (D.C.); 3 Office of the Texas State Chemist, Texas A&M AgriLife Research, Texas A&M University System, College Station, TX 77841, USA; kml@otsc.tamu.edu (K.-M.L.); tjh@otsc.tamu.edu (T.J.H.); 4 Department of Bio-Industrial Machinery Engineering, College of Agriculture and Life Science, Gyeongsang National University, 501 Jinju-daero, Jinju-si 52828, Republic of Korea

**Keywords:** aflatoxins, classification, fumonisins, hyperspectral imaging, maize

## Abstract

Aflatoxins and fumonisins, commonly found in maize and maize-derived products, frequently co-occur and can cause dangerous illness in humans and animals if ingested in large amounts. Efforts are being made to develop suitable analytical methods for screening that can rapidly detect mycotoxins in order to prevent illness through early detection. A method for classifying contaminated maize by applying hyperspectral imaging techniques including reflectance in the visible and near-infrared (VNIR) and short-wave infrared (SWIR) regions, and fluorescence was investigated. Machine learning classification models in combination with different preprocessing methods were applied to screen ground maize samples for naturally occurring aflatoxin and fumonisin as single contaminants and as co-contaminants. Partial least squares–discriminant analysis (PLS-DA) and support vector machine (SVM) with the radial basis function (RBF) kernel were employed as classification models using cut-off values of each mycotoxin. The classification performance of the SVM was better than that of PLS-DA, and the highest classification accuracies for fluorescence, VNIR, and SWIR were 89.1%, 71.7%, and 95.7%, respectively. SWIR imaging with the SVM model resulted in higher classification accuracies compared to the fluorescence and VNIR models, suggesting that as an alternative to conventional wet chemical methods, the hyperspectral SWIR imaging detection model may be the more effective and efficient analytical tool for mycotoxin analysis compared to fluorescence or VNIR imaging models. These methods represent a food safety screening tool capable of rapidly detecting mycotoxins in maize or other food ingredients consumed by animals or humans.

## 1. Introduction

Mycotoxins are secondary metabolites produced by various fungi and are considered natural contaminants of cereals, so their presence is unavoidable. The most common mycotoxins that can cause health effects when ingested by humans and livestock are aflatoxins (AF), ochratoxins, fumonisins (FM), zearalenone, nivalenol, deoxynivalenol, and fumonisins [1]. The contamination of food and feed with mycotoxins is a serious problem worldwide. A very recent extensive metadata analysis of over 500,000 samples found that the prevalence of mycotoxins could be as high as 60–80%, depending on the mycotoxin of concern, the analytical method used, and the equipment detection limit [2]. 

AF and FM, produced by pathogenic species of *Aspergillus* and *Fusarium* fungi, respectively, cause many problems in agricultural products worldwide [3,4]. AF is well known as a Group 1 carcinogen and FM is also considered a possible class 2B carcinogen (carcinogenic to humans, as classified by the International Agency for Research on Cancer). 

In general, chemical analysis methods such as high-performance liquid chromatography (HPLC), liquid chromatography–mass spectrometry (LC-MS/MS), and gas chromatography (GC) have been used for mycotoxin analysis [5,6]. In addition, rapid analysis methods such as the enzyme-linked immunosorbent assay (ELISA), lateral flow assays (LFAs), and biosensors have been developed and used [7]. Although these methods show excellent performance, they are time- and resource-consuming, and the use of chemicals during analysis can have negative impacts on both human health and the environment. Therefore, fast, reliable, and simple technologies with improved accuracy and differentiation capabilities are becoming important for food safety, where a fast quality assurance system for accurate safety management is essential.

In recent years, various spectroscopy and hyperspectral imaging techniques for analyzing food products for safety have been developed to replace standard chemical methods [8,9,10,11,12]. Hyperspectral imaging in particular has been studied in the field of food safety and quality evaluations because it can obtain both spatial and spectral information from a sample [11,13,14,15]. 

Recent spectroscopy and hyperspectral imaging studies have been conducted using grains to detect mycotoxins in maize [9,16,17,18], rather than ground samples. There are pros and cons to using grain and ground samples, depending on purpose. Methods for evaluating individual grains have the advantage of mitigating the cross-contamination of mycotoxins by removing mycotoxin-contaminated samples from the entire sample, as several studies have shown that AF and FM contamination in maize grain samples is not homogeneous [17,19,20]. For chemical analysis, a multiple-grain sample should be ground and mixed homogeneously to be representative of the entire sample. In addition, such chemical analysis is commonly used in regulatory laboratories and provides the basis for legal action.

Research on the detection of mycotoxins such as AF, FM, and deoxynivalenol using hyperspectral imaging technology is being conducted for various agricultural products, but the method for detecting a single mycotoxin was the main focus in previous studies [17,21,22]. Because some fungi can produce multiple mycotoxins simultaneously and agricultural products can be contaminated by several fungi at the same time, several mycotoxins can contaminate a crop at once. In particular, many studies on the simultaneous occurrence of AF and FM contamination have been reported [23,24,25].

Thus, the need for research on the simultaneous detection of AF and FM is increasing. The use of visible and near-infrared (VNIR) spectroscopy (400–2500 nm) has been investigated to evaluate maize grains for aflatoxigenic fungus and AF contamination [26], and multispectral reflectance using nine selected VNIR wavelengths has been investigated for sorting maize grains contaminated with AF and FM [9]. The use of e-nose sensors combined with lateral flow immunoassays has been investigated for detecting the co-occurrence of AF and FM in maize samples [27]. However, no previous work has been found for discriminating AF contamination, FM contamination, and co-contaminated samples in maize using hyperspectral imaging systems.

Therefore, this study focused on investigating hyperspectral imaging and classification methods to establish suitable models to screen out ground maize samples for single-contaminant and co-contaminant AF and FM for the early detection of contaminated samples in a safety control laboratory.

## 2. Results and Discussion

### 2.1. AF and FM Analysis

For a total of 228 ground maize samples, AF and FM were analyzed by HPLC and LC-MS/MS, respectively. The concentration ranged from 0.021 to 0.585 mg/kg and from 1.1 to 16.6 mg/kg for AF-contaminated and FM-contaminated maize, respectively. Other statistics of mycotoxin analysis with the concentration range of co-contaminated samples are shown in Table 1. The concentration ranges of AF and FM contaminations of the naturally contaminated samples used in this study were not broad. Nevertheless, the concentrations were well distributed and are considered suitable for developing a screening method for AF and FM detection in maize. 

### 2.2. Spectral Analysis

**Figure 1:**
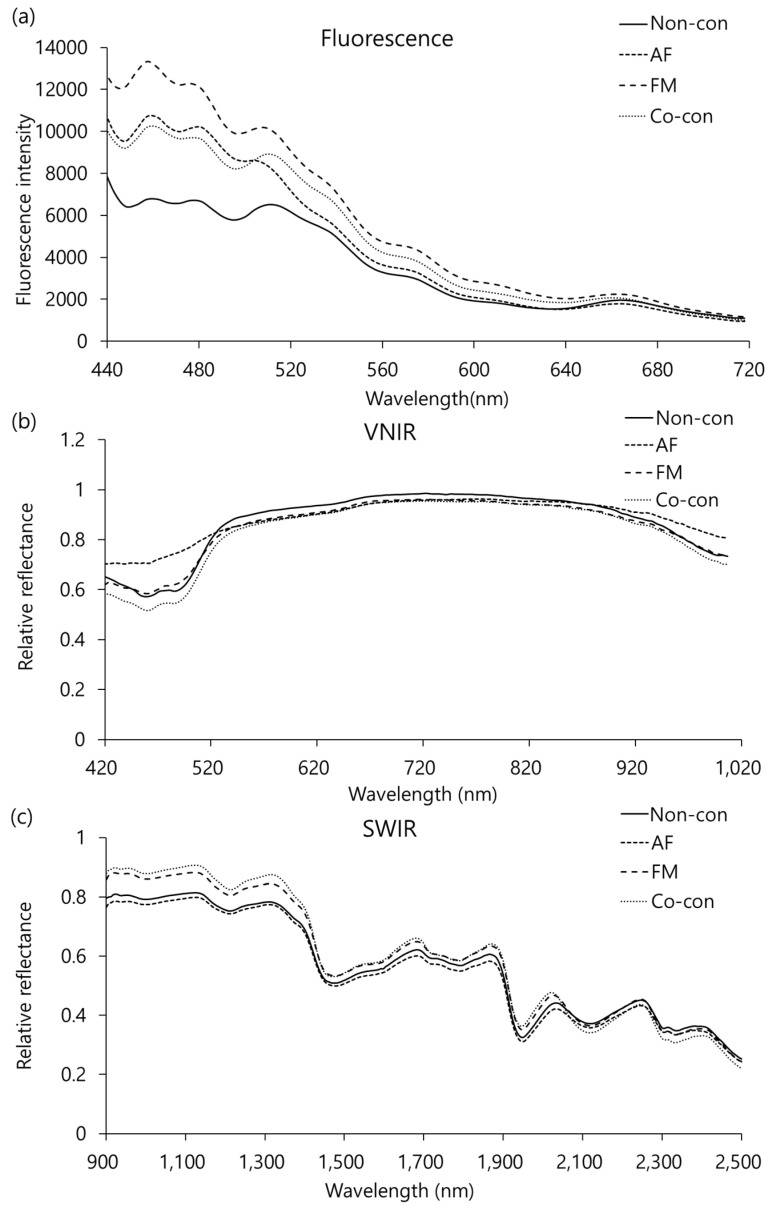
Mean raw spectra of four types of maize samples contaminated with mycotoxins. (**a**) Fluorescence, (**b**) VNIR reflectance, and (**c**) SWIR reflectance.

The mean spectra of ground maize samples and AF-contaminated, FM-contaminated, and AF+FM co-contaminated maize as calculated from the extracted fluorescence, VNIR, and short-wave infrared (SWIR) reflectance are shown in Figure 1. The mycotoxin concentration was not high compared to other organic compounds such as starch, protein, moisture, and fiber in the sample, so it could not be detected directly. For this reason, single-band comparisons did not reveal significant visual differences between aflatoxin-contaminated, fumonisin-contaminated, and co-contaminated maize (Figure 1). Therefore, multivariate methods such as machine learning and chemometric models can be applied to extract meaningful information correlated with mycotoxin concentrations from spectra.

Fluorescence peaks in the regions of 374–609 nm and 650–800 nm are related to AFB1 and oxidation substances [28] and pheophytins and chlorophylls [29,30]. The visible spectral region (400–700 nm) is primarily related to the presence of pigments such as chlorophyll and carotenoids that impart color to the sample [31]. The SWIR spectra showed higher-intensity peaks at 1320, 1870, and 2254 nm and higher-intensity valleys at 1208, 1474, and 1940 nm. The features at 1474 and 1870 nm are associated with O-H vibrations of starch [32], and those at 1895 nm correspond to the C-O-H and C-O-C angular deformation of carbohydrate in the fungal cell wall [33]. The 1208 nm feature may be attributed to the C-H stretching of CH_2_, related to fatty acid and oil [21,34]. The representative SWIR spectrum acquired through hyperspectral imaging indicates carbohydrate and lipids that provide a carbon source for fungal growth, and proteins that provide nitrogen sources [35]. 

### 2.3. Classification Result of AF, FM, and AM+FM Co-Contamination

The feasibility of using three hyperspectral imaging techniques for classifying mycotoxin-contaminated maize samples using partial least squares–discriminant analysis (PLS-DA) and support vector machine (SVM) with radial basis function (RBF) kernel models and cut-off values set at AF < 10 μg/kg and FM < 1 mg/kg was investigated. To evaluate the classification models, accuracy, precision, recall, and F1 scores were applied to nine pretreatment spectra (including raw spectra) for samples contaminated with mycotoxins (Tables S1–S3). In the sample used in the current study, when the values of calibration accuracy, validation accuracy, precision, recall, and F1 score according to the preprocessing method of the PLS-DA model and the SVM model were compared with each other, the performance of the SVM model was excellent in three hyperspectral imaging techniques (Tables S1–S3). Although the PLS-DA and SVM models gave different results, they are commonly used algorithms for mycotoxin detection in maize [22]. Previous studies have reported algorithms such as PLS-DA [11] and SVM [9,36] to show promising results in mycotoxin detection. 

The five best preprocessing methods with the SVM model in this study are detailed in Table 2. The results of the SVM model were excellent, with calibration and validation set accuracies of 97.3% and 89.1%, respectively, for fluorescence using standard normal variate (SNV) preprocessing; 81.3% and 71.7%, respectively, for VNIR using range normalization; and 98.4% and 95.7%, respectively, for SWIR using the Savitzky–Golay second derivative (SG2) (Table 2). As shown in Figure 1, since the spectra acquired by each hyperspectral imaging system were different, the best accuracy shown by the preprocessing was also different. Based on the results of all parameters such as calibration accuracy, validation accuracy, precision, recall, and F1 score, the use of SWIR with SG2 preprocessing showed the best results.

**Figure 2:**
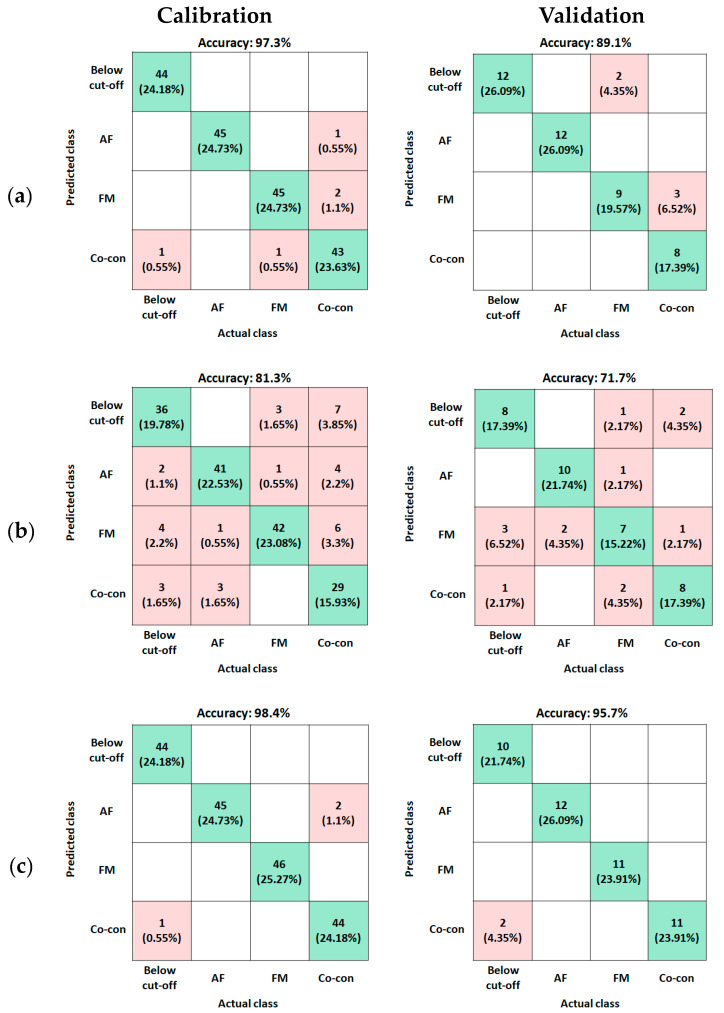
The multiclass confusion matrices of best classification results achieved for different hyperspectral imaging techniques for detecting mycotoxin contamination in ground maize using SVM model. (**a**) Fluorescence with SNV, (**b**) VNIR with range normalization, and (**c**) SWIR with SG2. Below cut-off: aflatoxin contents below 10 μg/kg and fumonisin contents below 1 mg/kg, AF: aflatoxin contamination, FM: fumonisin contamination, Co-con: both aflatoxin and fumonisin contamination.

The multiclass confusion matrix can be used to calculate classification statistics by comparing the actual and predicted classes, provide a visualization of the classification performance, and help in understanding what distinguishes each group (Figure 2). The rows and columns of the confusion matrix correspond to the predicted and actual class, respectively. The numbers in diagonal cells represent correctly predicted classes in actual classes, and the sum of the percentages of each diagonal cell is the accuracy of calibration and validation in different hyperspectral imaging techniques.

In comparing other evaluation criteria among the three types of imaging, the best classification results using validation data were obtained by the SWIR imaging method using max normalization and SG2, with 96.2% precision, 95.8% recall, and 95.6% F1 score (Table 2). Fluorescence with SVM models using SNV preprocessing showed 90.2% precision, 88.6% recall, and 88.7% F1 score, which was noticeably better than the VNIR scores of 72.6% precision, 71.6% recall, and 71.9% F1 score. 

Many previous studies have used hyperspectral imaging and spectroscopy techniques to quantitatively or qualitatively analyze the presence of a single mycotoxin in contaminated food and agricultural products such as AF in maize [26,36] and peanut [37], FM in maize [17,38], ochratoxin A in wheat [39], deoxynivalenol in wheat [21], and zearalenone in maize [38,40]. Some studies involving both single and co-contaminated maize samples were also performed but used other non-imaging methods such as e-nose and lateral flow immunoassays [27]. As mentioned in the introduction, the simultaneous occurrence of AF and FM is frequent. However, there are not many studies published related to it. Therefore, this study was performed by investigating and providing ground maize samples with AF-, FM-, and co-contaminated samples at various concentrations. 

Comparing the classifications achieved from using the fluorescence, VNIR, and SWIR imaging systems, the SWIR reflectance results showed higher accuracy, precision, recall, and F1 scores than those of the others, suggesting that SWIR with the SVM model algorithm may perform better than the fluorescence and VNIR algorithms for rapid analysis of mycotoxin detection in maize samples (Figure 2 and Table 1). Machine learning results indicated that classification among maize samples with various mycotoxins was possible based on the spectra acquired by the SWIR hyperspectral imaging system.

## 3. Conclusions

A rapid detection method based on using hyperspectral fluorescence, VNIR, or SWIR imaging combined with machine learning algorithms for the detection of single-contaminated and co-contaminated AF and FM in ground maize samples has been proposed and validated in the present work. The SWIR with SVM model showed higher accuracy and generalization performance. Existing studies using hyperspectral imaging systems focus on the development of methods for determining a single mycotoxin contamination. However, this study was conducted because of the high incidence of simultaneous mycotoxin contaminants in maize, and further studies on the simultaneous detection of other mycotoxins, such as ochratoxin A, zearalenone, and deoxynivalenol contamination, are needed. 

## 4. Materials and Methods 

### 4.1. Sample Preparation

A total of 228 maize samples naturally contaminated with either AF or FM, or both AF and FM, were provided by the Office of Texas State Chemist (OTSC). These regulatory maize samples used for feed were collected by the Texas Feed and Fertilizer Control Service. The maize samples used in the study spanned a range of varieties and qualities because they were collected from several feed companies located in Texas. The statistical information of the samples’ mycotoxin concentrations is summarized in Table 1. The maize samples were ground using a Retsch Ultra Centrifugal Mill ZM 200 (Retsch, Haan, German) with a 0.75 mm diameter screen. The polyethylene bottles were used to store ground samples at 4 °C until further analysis. 

### 4.2. Mycotoxin Measurement

#### 4.2.1. AF Analysis Using HPLC

A 50 g ground maize subsample was ground and extracted by placement in 250 mL of methanol/water (70:30, *v*/*v*) for shaking for 1 h at 200 rpm. The extract was filtered through paper; diluted at a ratio of 1:6 with deionized water; and then, after the addition of 1 g of NaCl, placed into an Aflatest TM immunoaffinity column (Vicam, Watertown, MA, USA). After the column was washed with water, aflatoxin was eluted from the column with 1 mL of methanol. The elute was then diluted with HPLC water and injected into the HPLC system (Waters 2695, Milford, MA, USA). The HPLC system consisted of a Waters model 2695 autosampler, tube-and-shell membrane reactor module, a Waters 2475 Multi λ fluorescence detector set at 360 nm for excitation and 420 nm for emission, a Waters model 746 data module integrator, and a Spherisorb column (4.6 by 150 mm) protected with a guard column (4.6 by 10 mm). The mobile phase was a mixture of water:acetonitrile:methanol (3:1:1) at a flow rate of 1.0 mL/min. Sample data were collected and analyzed using the Empower software (Milford, MA, USA). A combined AF standards mixture was prepared by mixing aflatoxins B1, B2, G1, and G2. The mixture was dried under nitrogen at room temperature, dissolved with methanol, and added to an autosampler vial for injection to the HPLC. 

#### 4.2.2. FM Analysis Using LC-MS/MS

A 5 g portion of ground maize sample was extracted with 100 mL of methanol/water (70:30, *v*/*v*) using a mechanical shaker for 15 min. A 25 mL volume of the extract was then centrifuged at 3000 rpm for 5 min. The samples with high FM concentration were further diluted with 50% methanol in water after centrifugation. A 40 µL volume of (U-[13C34]-FB1) solution (250 ng mL^−1^) was added to 1 mL of the centrifuged supernatant. The solution spiked with the internal standard was then forced into a PVDF 0.2 μm syringe filter prior to LC-MS/MS analysis. The chromatographic separation of FM was carried out using a Waters UPLC Quattro Premier XE system (Waters, Milford, MA, USA) equipped with a BEH C18 column (2.1 mm × 50 mm, 1.7 µm particle size) and an electrospray interface (ESI). The mobile phases A and B were water with 0.1% formic acid and methanol with 0.1% formic acid, respectively. The mobile phases at a flow rate of 0.3 mL/min were applied in the following gradient conditions: at 0 min, 50% A:50% B; at 2.5 min, 10% A:90% B; at 2.6 min, 0% A:100% B; at 3.6 min, 50% A:50% B; at 5.0 min (end), 50% A:50% B. The positive electrospray ionization (ESI) mode was selected at a source temperature of 140 °C, a desolvation temperature of 400 °C, a cone voltage of 50 V, and a desolvation gas (nitrogen) flow of 600 L/h. Multiple reaction monitoring (MRM) was used for MS analysis by employing high-purity argon as a collision gas at a pressure of 4.7 × 10^−3^ mbar. The precursor ions used for detection of FM were *m*/*z* 722 for FB1 and *m*/*z* 706 for FB2/FB3. Likewise, the product ions of *m*/*z* 334.4, *m*/*z* 318.4, and *m*/*z* 336.4 were used for detection of FB1, FB2, and FB3, respectively. 

### 4.3. Spectroscopy and Spectral Acquisition

Line-scan imaging systems developed in-house to conduct fluorescence, visible and near-infrared (VNIR) reflectance, and short-wave infrared (SWIR) reflectance imaging were used to acquire hyperspectral data from the ground maize samples. Detailed information regarding the systems were reported in our previous study [41]. Briefly, reflectance and fluorescence images were acquired in the spectral regions of 419–1007 nm (125 bands) and 438–718 nm (60 bands), respectively, using a VNIR hyperspectral system equipped with a 150 W quartz tungsten halogen lamp (Dolan Jenner, Boxborough, MA, USA) and two UV line lights, each with four 10 W 365 nm light-emitting diodes (LEDs) (LedEngin, San Jose, CA, USA) [42]. The detection unit consisted of a 23 mm focal-length lens, a 14-bit electron-multiplying charge-coupled-device (EMCCD) camera (Luca DL 604M, And or Technology, South Windsor, CT, USA), and an imaging spectrometer (Hyperspec-VNIR, Headwall Photonics, Fitchburg, MA, USA). The SWIR hyperspectral system consisted of a custom-designed two-unit lighting system with four 150 W gold-coated halogen lamps with MR16 reflectors [15]. The detection unit included a 25 mm focal-length lens, a hyperspectral camera module including a 16-bit mercury cadmium telluride (MCT) array detector, and an imaging spectrograph (Hyperspec-SWIR, Headwall Photonics, Fitchburg, MA, USA). Images were acquired in a corresponding wavelength range of 1007–2472 nm (249 bands).

### 4.4. Spectral Data Preprocessing

VNIR and SWIR hyperspectral images were calibrated using a flat-field calibration method to extract the standardized spectral response of the samples, and fluorescence images were calibrated for spatial calibration to account for spatial intensity variations of individual LEDs [43] in a linear UV-A illumination unit. 

The region of interest (ROI) selection was performed to extract spectral data from each hyperspectral image after correcting all hyperspectral images, and the mean spectrum of each sample was extracted from the corresponding ROI and used for further data analysis.

Preprocessing of hyperspectral data is an important step before modeling because the raw spectral data contain interference signals such as noise, baseline shift, surface heterogeneity, particle deviation, and light scattering. Thus, eight different pretreatment methods were used to process the mean spectral data of ground maize samples: normalization (max, mean, range), multiplicative scatter correction (MSC), SNV, Savitzky–Golay 1st derivative (SG1) and 2nd derivative (SG2), and smoothing with 3 windows. Image correction, spectral extraction, preprocessing, and modeling were all performed using programs developed in MATLAB (MathWorks, Natick, MA, USA).

### 4.5. Development and Evaluation of Classification Models

A linear multivariate model, PLS-DA, and a nonlinear model, SVM with RBF kernel, were used to develop classification models. PLS-DA is a predictive model based on the classical PLS regression method with advantages such as noise reduction and variable selection [44,45]. The SVM is a model with the advantage of avoiding local minima as a generalization function [46,47]. The RBF is one of the classical kernel functions of SVM models and has been widely used because of its ability to reduce the computational complexity of the training procedure and to handle nonlinear relationships between spectral and target attributes [48].

Preprocessed spectral data from ground maize samples were used to develop a classification model for multi-mycotoxin-contaminated samples. When creating the classification models, a leave-one-out cross-validation method was performed to increase the generalization of the model and prevent overfitting.

The samples were classified to 4 groups: Group 1 for non-contaminated (AF and FM concentrations below 10 μg/kg and 1 mg/kg, respectively), Group 2 for AF contamination, Group 3 for FM contamination, and Group 4 for co-contamination of AF and FM. Each group contained 57 samples. Four groups of 57 sample spectra (228 samples in total) were randomly divided into a calibration set and a validation set (below cut-off and AF-contaminated group divided 45 calibration samples to 12 validation samples, FM and co-contaminated group divided 46 to 11) for developing and testing the classification models (Table 1). Cut-off values were set at 10 μg/kg for AF and 1 mg/kg for FM, which were well below regulatory limits. The calibration and validation accuracy, precision, recall, and F1 score were calculated using the following equations:

$$Accuracy\left(\%\right)=\frac{TP+TN}{TP+TN+FP+FN}\times 100$$

$$Precision\left(\%\right)=\frac{TP}{TP+FP}\times 100$$

$$Recall(\%)=\frac{TP}{TP+FN}\times 100$$

$$F1\;score(\%)=2\times \frac{Precision\times Recall}{Precision+Recall}\times 100$$

where TP is the number of true positive samples, FP is the number of false positive samples, TN is the number of true negative samples, FN is the number of false negative samples.

The image correction, spectral extraction, preprocessing, and all chemometric modeling were performed using programs developed in MATLAB (MathWorks, Natick, MA, USA).

## Figures and Tables

**Table 1:** Descriptive statistics of mycotoxin content in maize samples.

Parameter	No. of Samples	Min (mg/kg)		Max (mg/kg)		Median (mg/kg)		Mean (mg/kg)		SD (mg/kg)	
Below cut-off	57	AF < 0.01, FM < 1									
AF contaminated	57	0.021		0.585		0.056		0.158		0.183	
FM contaminated	57	1.1		16.6		4.2		4.8		2.8	
Co-contaminated	57	AF	FM	AF	FM	AF	FM	AF	FM	AF	FM
		0.012	1.1	0.136	15	0.042	4.4	0.049	5.1	0.028	2.8

**Table 2:** Chemometrics results of the SVM model for mycotoxin-contaminated samples analyzed by three hyperspectral imaging systems combined with different preprocessing methods.

Preprocessing Method			Raw	Max Normalization	Range Normalization	SNV	SG2
Fluorescence	Calibration (%)	Accuracy	50.5	92.9	92.9	97.3	100
	Validation (%)	Accuracy	39.1	80.5	87.0	89.7	67.4
		Precision	29.1	81.1	87.1	90.2	71.7
		Recall	40.9	80.1	86.6	88.6	67.6
		F1 score	29.6	80.0	86.2	88.7	67.9
VNIR	Calibration (%)	Accuracy	87.9	85.9	81.3	99.5	79.1
	Validation (%)	Accuracy	63.0	71.7	71.7	67.4	47.8
		Precision	62.5	71.2	72.6	67.7	48.8
		Recall	63.1	71.4	71.6	67.0	47.5
		F1 score	62.7	71.1	71.9	67.1	47.6
SWIR	Calibration (%)	Accuracy	97.8	92.0	87.4	100	98.4
	Validation (%)	Accuracy	89.1	95.7	87.0	91.3	95.7
		Precision	90.8	96.2	91.2	92.1	96.2
		Recall	88.8	95.8	86.7	91.3	95.8
		F1 score	89.0	95.6	86.8	91.5	95.6

## Data Availability

The data presented in this study are available on request from the corresponding author.

## Supplementary Materials

The following supporting information can be downloaded at: https://www.mdpi.com/article/10.3390/toxins15070472/s1, Table S1: Chemometrics results of the PLS-DA and SVM model for mycotoxin-contaminated samples analyzed by fluorescence combined with different preprocessing methods; Table S2: Chemometrics results of the PLS-DA and SVM model for mycotoxin-contaminated samples analyzed by VNIR combined with different preprocessing methods; Table S3: Chemometrics results of the PLS-DA and SVM model for mycotoxin-contaminated samples analyzed by SWIR combined with different preprocessing methods.
