# Supplementary material for: Rapid Detection of Single- and Co-Contaminant Aflatoxins and Fumonisins in Ground Maize Using Hyperspectral Imaging Techniques
Source: Toxins (Basel). 2023 Jul 22;15(7):472. doi: 10.3390/toxins15070472 (PMC10467122; doi:10.3390/toxins15070472)
Supplement: Supplementary file 1 [file toxins-15-00472-s001.zip › toxins-2471156-supplementary.pdf]

toxins

MDPI

Article

Rapid Detection of Single- and Co-contaminant Aflatoxins and Fumonisins in Ground Maize using Hyperspectral Imaging Techniques

Yong-Kyoung Kim [1], Insuck Baek [2], Kyung-Min Lee [3], Geonwoo Kim [2,4], Seyeon Kim [1], Sung-Youn Kim [1], Diane Chan [2], Timothy J. Herrman [3], Namkuk Kim [1,*] and Moon S. Kim [2,*]

[1] Division of Safety Analysis, Experiment & Research Institute, National Agricultural Products Quality Management Service, Gimcheon 39660, Republic of Korea; ykkim79@korea.kr (Y.-K.K.); syesther1114@korea.kr (S.K.); youn5326@korea.kr (S.-Y.K.)

[2] Environmental Microbial and Food Safety Laboratory, Agricultural Research Service, U.S. Department of Agriculture, Powder Mill Rd., Building 303 BARC-East, Beltsville, MD 20705, USA; insuck.baek@usda.gov (I.B.); geonwookim@gnu.ac.kr (G.K.); diane.chan@usda.gov (D.C.)

[3] Office of the Texas State Chemist, Texas A&M AgriLife Research, Texas A&M University System, College Station, TX 77841, USA; kml@otsc.tamu.edu (K.-M.L.); tjh@otsc.tamu.edu (T.J.H.)

[4] Department of Bio-industrial Machinery Engineering, College of Agriculture and Life Science, Gyeongsang National University, 501 Jinju-daero, Jinju-si 52828, Republic of Korea

* Correspondence: nkvirus@korea.kr (N.K.); moon.kim@usda.gov (M.S.K.); Tel.: +82-54-429-7766 (N.K.); +1-301-504-8462 (M.S.K.)

Table S1. Chemometrics results of the PLS-DA and SVM model for mycotoxin-contaminated samples analyzed by Fluorescence combined with different preprocessing methods.

Preprocessing Method	PLS-DA					SVM				
	Calibration (%)	Validation (%)				Calibration (%)	Validation (%)			
	Accuracy	Accuracy	Precision	Recall	F1 score	Accuracy	Accuracy	Precision	Recall	F1 score
**Raw**	52.2	32.6	48.6	32.6	34.9	50.5	39.1	29.1	40.9	29.6
**Max**	58.3	39.1	52.8	39.4	41.3	92.9	80.4	81.1	80.1	80.0
**Mean**	57.7	37.0	51.3	37.1	39.5	95.1	26.1	6.5	25.0	10.3
**Range**	57.7	37.0	46.6	37.3	39.1	92.9	87.0	87.1	86.6	86.2
**SNV**	39.2	32.6	41.7	32.6	28.0	97.3	89.1	90.2	88.6	88.7
**MSC**	39.0	32.6	41.7	32.6	28.0	50.5	26.1	6.5	25.0	10.3
**Smoothing**	51.6	30.4	44.5	30.3	32.0	50.5	41.3	32.2	43.2	32.3
**SG1**	42.3	32.6	39.3	32.2	30.3	50.5	37.0	26.2	38.6	27.9
**SG2**	43.7	34.8	40.6	34.5	32.0	100	67.4	71.7	67.6	67.9

*Precision, Recall, F1 scores are average value of each groups.

Table S2. Chemometrics results of the PLS-DA, SVM model for mycotoxin-contaminated samples analyzed by VNIR combined with different preprocessing methods.

Preprocessing Method	PLS-DA					SVM				
	Calibration (%)	Validation (%)				Calibration (%)	Validation (%)			
	Accuracy	Accuracy	Precision	Recall	F1 score	Accuracy	Accuracy	Precision	Recall	F1 score
**Raw**	31.3	28.3	38.6	28.4	21.4	87.9	63.0	62.5	63.1	62.7
**Max**	30.2	23.9	12.3	24.1	16.1	85.9	71.7	71.2	71.4	71.1
**Mean**	29.7	23.9	12.0	24.1	15.9	100	26.1	6.5	25.0	10.3
**Range**	28.6	21.7	11.0	22.0	14.6	81.3	71.7	72.6	71.6	71.9
**SNV**	20.3	19.6	9.2	20.1	12.2	99.5	67.4	67.7	67.0	67.1
**MSC**	24.7	23.9	23.9	24.2	18.6	99.5	26.1	6.5	25.0	10.3
**Smoothing**	31.3	30.4	39.7	30.7	22.8	90.1	60.9	61.7	61.0	61.1
**SG1**	29.1	26.1	13.0	26.3	17.3	100	50.0	47.9	49.2	48.4

**SG2**	28.6	34.8	40.6	34.5	32.0	79.1	67.4	71.7	67.6	67.9

Table S3. Chemometrics results of the PLS-DA, SVM model for mycotoxin-contaminated samples analyzed by SWIR combined with different preprocessing methods.

**Preprocessing Method**	**PLS-DA**					**SVM**				
	**Calibration (%)**	**Validation (%)**				**Calibration (%)**	**Validation (%)**			
	**Accuracy**	**Accuracy**	**Precision**	**Recall**	**F1 score**	**Accuracy**	**Accuracy**	**Precision**	**Recall**	**F1 score**
**Raw**	58.2	56.5	65.1	56.4	57.3	97.8	89.1	90.8	88.8	89.0
**Max**	58.8	60.9	77.0	60.6	57.4	91.8	95.7	96.2	95.8	95.6
**Mean**	53.3	58.7	68.9	58.5	58.2	26.1	26.1	6.5	25.0	10.3
**Range**	54.9	50.0	63.9	49.8	49.9	87.4	87.0	91.2	86.7	86.8
**SNV**	59.9	54.3	66.8	54.2	53.3	100	91.3	92.1	91.3	91.5
**MSC**	57.1	54.3	66.8	54.2	53.3	100	23.9	6.0	25.0	9.6
**Smoothing**	59.9	52.2	60.1	52.1	52.5	96.7	89.1	90.8	88.8	89.0
**SG1**	59.3	47.8	61.7	47.5	50.4	100	95.7	95.8	95.6	95.6
**SG2**	59.9	34.8	40.6	34.5	32.0	98.4	67.4	71.7	67.6	67.9
